# Trends in unhealthy lifestyle factors in US NHANES respondents with cardiovascular disease for the period between 1999 and 2018

**DOI:** 10.3389/fcvm.2023.1169036

**Published:** 2023-05-19

**Authors:** Yanting Liang, Fengyao Liu, Han Yin, Xiaohe Shi, Yilin Chen, Haochen Wang, Yu Wang, Bingqing Bai, Yuting Liu, Quanjun Liu, Chao Wu, Xueju Yu, Huan Ma, Qingshan Geng

**Affiliations:** ^1^ Department of Cardiology, Guangdong Provincial People's Hospital (Guangdong Academy of Medical Sciences), Southern Medical University, Guangzhou, China; ^2^ Department of Cardiology, Shenzhen People's Hospital, Jinan University, Shenzhen, China; ^3^ School of Medicine South China University of Technology, Guangzhou, China; ^4^ Guangdong Provincial Geriatrics Institute, Guangdong Provincial People's Hospital (Guangdong Academy of Medical Sciences), Southern Medical University, Guangzhou, China; ^5^ School of Biomedical Sciences and Engineering, South China University of Technology, Guangzhou, China

**Keywords:** unhealthy lifestyle factors, cardiovascular disease, national health and nutrition examination survey, trend, United States

## Abstract

**Objectives:**

To examine national trends in unhealthy lifestyle factors among adults with cardiovascular disease (CVD) in the United States (US) between 1999 and 2018.

**Methods:**

We analyzed data from National Health and Nutrition Examination Survey (NHANES), a nationally representative survey of participants with CVD who were aged ≥20 years, which was conducted between 1999 and 2000 and 2017–2018. CVD was defined as a self-report of congestive heart failure, coronary heart disease, angina, heart attack, or stroke. The prevalence rate of each unhealthy lifestyle factor was calculated among adults with CVD for each of the 2-year cycle surveys. Regression analyses were used to assess the impact of sociodemographic characteristics (age, sex, race/ethnicity, family income, education level, marital status, and employment status).

**Results:**

The final sample included 5610 NHANES respondents with CVD. The prevalence rate of their current smoking status remained stable among respondents with CVD between 1999 and 2000 and 2017–2018. During the same period, there was a decreasing trend in the age-adjusted prevalence rate of poor diet [primary American Heart Association (AHA) score <20; 47.5% (37.9%–57.0%) to 37.5% (25.7%–49.3%), *p* < 0.01]. Physical inactivity marginally increased before decreasing, with no statistical significance. The prevalence rate of sedentary behavior increased from 2007 to 2014 but subsequently returned to its original level in 2018 with no statistical significance. The age-adjusted prevalence rate of obesity increased from 32% (27.2%–36.8%) in 1999–2000 to 47.9% (39.9%–55.8%) in 2017–2018 (*p* < 0.001). The age-adjusted prevalence rate of depression increased from 7% (4.2%–9.9%) in 1999–2000 to 13.9% (10.2%–17.6%) in 2017–2018 (*p* = 0.056). Trends in mean for each unhealthy lifestyle factor were similar after adjustment for age. We found that respondents who had low education and income levels were at a higher risk of being exposed to unhealthy lifestyle factors (i.e., smoking, poor diet, and physical inactivity) than those who had high education and income levels.

**Conclusions:**

There is a significant reduction in the prevalence rate of poor diet among US adults with CVD between 1999 and 2018, while the prevalence rate of obesity showed increasing trends over this period. The prevalence rate of current smoking status, sedentary behavior, and depression was either stable or showed an insignificant increase. These findings suggest that there is an urgent need for health policy interventions targeting unhealthy lifestyles among adults with CVD.

## 1. Introduction

Cardiovascular disease (CVD) is a major cause of morbidity, mortality, and healthcare expenditure worldwide. Previous research has reported a 30.8% decline in mortality rates among United States (US) patients with CVD between 2000 and 2011, with stable rates found between 2010 and 2018 (1–3). How this is influenced by lifestyle change is unclear; however, the risk of developing a major non-communicable disease, including cardiovascular disease, is known to be critically influenced by lifestyle choices. Unfavorable lifestyle choices are associated with higher risks of all-cause and CVD mortality (4). According to previous literature reviews and national guidelines, CVD could be reduced by addressing unhealthy lifestyle factors such as smoking, physical inactivity, sedentary behavior, poor diet, and obesity (5–8).

Accumulating evidence also demonstrates that mental health conditions, including depression, are associated with a higher risk of CVD (9, 10).

Previous studies have generally examined the trends in cardiovascular risk factors such as blood pressure, total cholesterol, and glycated hemoglobin among the general population (11–14). Cheng et al. found that the proportion of adherence to the physical activity guidelines (PAG) among US adults with a history of CVD increased from 2007 to 2008 to 2017–2018 (15). There are a few reports about the trends in other unhealthy lifestyle factors over time in US adults with CVD. This information would help improve the evaluation of the effectiveness of historical public health policies and guide future disease prevention and health promotion strategies.

This study used data from the US National Health and Nutrition Examination Survey (NHANES) to examine the trends in unhealthy lifestyle factors in adults with CVD between 1999 and 2018. We also examined the risk for unhealthy lifestyle factors among different sociodemographic groups to address those who are most at risk.

## 2. Methods

### 2.1. Data source

The US NHANES is a multistage, nationally representative cross-sectional survey of the civilian, non-institutionalized population conducted in 2-year cycles. In each survey, data are collected on demographics, socioeconomic status, physical examination findings, laboratory results, health conditions, and behaviors of respondents during in-home interviews and at mobile examination centers. Data on age, race and ethnicity, sex, education, income, and medical history are collected using a standardized questionnaire during the in-house interview.

The National Center for Health Statistics Research Ethics Review Board approved the study and written informed consent was obtained from respondents.

### 2.2. Study population

For the current analysis, data from the 10 surveys conducted between 1999 and 2000 and 2017–2018 were utilized. CVD was defined if the respondents reported being told by a doctor or another health professional that they had a diagnosis of congestive heart failure, coronary heart disease, angina, heart attack, or stroke. Respondents with cardiovascular disease were included in the analysis, while those who were pregnant, breastfeeding, aged <20 years, or did not complete the NHANES physical examination were excluded. A total of 5,610 respondents were included.

### 2.3. Sociodemographic characteristics

Sex was defined as male or female. Age was grouped as 20–44, 45–64, or ≥65 years. Race/ethnicity was self-reported and categorized as non-Hispanic White, non-Hispanic Black, Hispanic, or other (including Non-Hispanic Asian, other Hispanic, Multi-Racial, and other). Education level was classified as below high school (considered low education), high school graduate or General Equivalent Diploma (GED), or some college or above. The income-to-poverty ratio (PIR) was used as a measure of family income, with outcomes grouped as <1.30 (considered low income), 1.30–3.49, or ≥3.50. Marital status was categorized as married or living with partner, never married, and widowed/divorced/separated. Employment status was either unemployed or employed.

### 2.4. Unhealthy lifestyle factors

The unhealthy lifestyle factors of interest included current status of smoking, obesity, physical inactivity, sedentary behavior, depression, and unhealthy diet. A respondent was considered a current smoker if they had smoked ≥100 cigarettes over their lifetime and currently smoked cigarettes every day/some days or had used tobacco/nicotine in the last 5 days. A body mass index (BMI) ≥30 kg/m^2^ was considered to indicate obesity. Depression was defined by a score ≥10 on the Patient Health Questionnaire-9 (PHQ-9) (16). These data were available from the 2005–2018 survey.

Moderate- and vigorous-intensity aerobic physical activity was recorded in the Global Physical Activity Questionnaire (GPAQ), including questions on work-related, leisure-time, and transportation-related activities during the past 30 days. Respondents were classified as physically inactive if they did not adhere to the PAG for aerobic activity (17). We defined physical inactivity in respondents if they participated in <150 min/week of moderate-intensity physical activity, or <75 min/week of vigorous-intensity physical activity, or an equivalent combination of the two. With regard to sedentary behavior, respondents were asked to report the time they spent on a typical day sitting at school, at home, getting to and from places, or with friends including time spent sitting at a desk, traveling in a car or bus, reading, playing cards, watching television, or using a computer. Sedentary behavior was defined as sitting for longer than 6 h/day (18). Physical activity and sitting durations were available only from the 2007–2008 surveys.

Dietary intake was assessed in NHANES using 24-h dietary recalls. We created a diet score based on the American Heart Association (AHA) 2020 Strategic Impact Goals for diet, which have been associated with cardiovascular outcomes (19). AHA score components include: (1) primary: total fruits and vegetables, whole grains, fish and shellfish, sugar-sweetened beverages, and sodium; and (2) secondary: adding nuts, seeds, legumes, processed meat, and saturated fat. The diet score was constructed by summing all components. In our study, poor diet was defined as achieving <20 (total possible points = 500 for the primary AHA score or achieving <32 (total possible points = 800 for the secondary AHA score.

Because data were not available for all factors between 1999 and 2006, we assessed the number of unhealthy lifestyle factors in each respondent from 2007–2008 to 2017–2018. A respondent was given 1 point for unhealthy lifestyle factors, up to a maximum of 6 points. The unhealthy lifestyles score was classified into three levels: ideal (0 points), intermediate (1–2 points), or poor (3–6 points).

### 2.5. Statistical analysis

Age-standardized estimates for each sociodemographic variable and unhealthy lifestyle factor were calculated for respondents with CVD in each survey. A summary of specific characteristics was presented as means and percentages for continuous and categorical variables, respectively, with 95% confidence interval (95% CI). The annual percentage change (APC) and average annual percentage change (AAPC) were analyzed by using joinpoint regression, which showed trends in unhealthy lifestyle factors over the surveys. We also assessed the distribution of the number of unhealthy lifestyle factors in each survey. Odds ratios (ORs) of an unhealthy lifestyle factor were evaluated using logistic regression models. All models were first adjusted for age, sex, and race/ethnicity and then additionally for socioeconomic factors (family income, education level, marital status, and employment status).

All analyses were conducted with the use of R version 3.6.3 (R Foundation for Statistical Computing) and Joinpoint Regression Program version 4.8.0.1, using the recommended sample weights that account for an oversampling of certain populations and survey non-response (20). A two-sided *p* < 0.05 was considered to be statistically significant.

## 3. Results

### 3.1. Sample characteristics

The final analytic sample included 5,610 respondents with CVD in the 1999–2018 NHANES. The demographic profile remained comparable over the course of the surveys (1999–2018) in terms of sex, race/ethnicity, employment status, and marital status. There were reductions in the proportion of participants with a below high school education (35.6% in 1999–2000 to 16.6% in 2017–2018), and increasing proportions of respondents with some college education or above (38.5%–56.1%) and aged ≥65 years (42.1%–49.9%). There were also trends for gradually increasing family income, with proportions of respondents with a low income (PIR < 1.30) gradually reducing, while the proportion with middle (1.30–3.49) and high (≥3.50) incomes gradually increased. Details on the characteristics of the participants are provided in Table 1.

**Table 1:** Demographics of adult NHANES respondents with cardiovascular disease for the period between 1999 and 2018.

	NHANES survey^a^									
Characteristic, % (95% CI)	1999–2000	2001–2002	2003–2004	2005–2006	2007–2008	2009–2010	2011–2012	2013–2014	2015–2016	2017–2018
	*n* = 460	*n* = 525	*n* = 585	*n* = 521	*n* = 655	*n* = 624	*n* = 507	*n* = 564	*n* = 606	*n* = 563
Age group (years)										
20–44	17.3 (12.7–21.8)	21.2 (15.2–27.2)	12.6 (9.1–16.1)	19.2 (12.7–25.7)	17.5 (12.2–22.9)	17.5 (12.7–22.4)	21.2 (16.5–25.9)	16.9 (11.9–22.0)	17.8 (13.7–21.9)	9.7 (6.1–13.3)
45–64	40.6 (36.6–44.6)	35.9 (26.8–45.0)	37.0 (30.5–43.5)	34.6 (29.6–39.6)	37.9 (33.0–42.9)	37.8 (33.6–42.0)	30.9 (24.3–37.5)	32.4 (27.4–37.4)	33.4 (26.9–39.9)	40.4 (33.3–47.5)
≥65	42.1 (38.3–46.0)	42.9 (35.6–50.2)	50.4 (44.9–56.0)	46.2 (40.8–51.6)	44.5 (40.0–49.1)	44.7 (40.1–49.2)	47.9 (40.2–55.6)	50.6 (44.8–56.5)	48.8 (42.3–55.2)	49.9 (43.4–56.4)
Sex										
Male	54.2 (47.4–61.0)	53.5 (49.0–58.1)	54.2 (49.2–59.3)	54.2 (49.2–59.2)	53.1 (48.4–57.9)	59.8 (55.0–64.6)	53.3 (48.2–58.4)	52.8 (46.9–58.6)	51.8 (47.6–56.0)	61.5 (55.2–67.8)
Female	45.8 (39.0–52.6)	46.5 (41.9–51.0)	45.8 (40.7–50.8)	45.8 (40.8–50.8)	46.9 (42.1–51.6)	40.2 (35.4–45.0)	46.7 (41.6–51.8)	47.2 (41.4–53.1)	48.2 (44.0–52.4)	38.5 (32.2–44.8)
Race/ethnicity										
Non-Hispanic White	78.9 (72.3–85.4)	78.9 (71.7–86.1)	82.4 (78.2–86.6)	80.2 (74.0–86.4)	73.6 (66.8–80.3)	75.6 (68.8–82.4)	72.9 (67.0–78.7)	75.0 (69.3–80.7)	69.1 (62.3–76.0)	72.5 (65.7–79.4)
Non-Hispanic Black	8.0 (5.0–10.9)	11.5 (6.4–16.6)	7.9 (5.2–10.7)	11.6 (7.5–15.8)	10.6 (7.2–14.0)	10.7 (7.2–14.2)	10.4 (6.7–14.2)	10.0 (7.4–12.5)	11.2 (6.8–15.5)	8.9 (5.2–12.7)
Hispanic	9.3 (3.0–15.6)	7.8 (1.2–14.5)	4.6 (1.5–7.6)	5.1 (2.6–7.7)	8.6 (5.3–11.9)	9.1 (4.1–14.0)	9.9 (4.8–15.0)	9.3 (6.1–12.5)	10.4 (6.1–14.7)	9.0 (5.9–12.2)
Other^b^	3.9 (0.6–7.1)	1.8 (−0.2–3b.8)	5.1 (2.1–8.1)	3.0 (1.1–4.9)	7.2 (4.0–10.4)	4.6 (2.2–7.0)	6.8 (3.5–10.0)	5.8 (3.1–8.5)	9.3 (5.5–13.2)	9.5 (4.8–14.2)
Family income (PIR)										
<1.30	27.3 (21.5–33.1)	28.6 (22.3–34.9)	23.5 (17.2–29.9)	21.4 (17.3–25.5)	22.2 (18.7–25.7)	27.5 (22.0–33.1)	27.5 (22.0–33.1)	28.3 (23.4–33.3)	23.7 (18.6–28.7)	16.9 (13.6–20.1)
1.30–3.49	33.6 (27.4–39.8)	33.1 (28.1–38.2)	39.1 (35.2–43.0)	43.2 (38.2–48.2)	35.3 (31.4–39.2)	34.7 (28.4–41.1)	34.7 (28.4–41.1)	39.6 (33.5–45.6)	35.5 (29.2–41.9)	38.3 (30.3–46.2)
≥3.50	31.0 (24.8–37.1)	32.3 (27.0–37.5)	33.1 (26.6–39.5)	29.1 (22.9–35.3)	32.2 (28.0–36.4)	30.3 (19.6–41.0)	30.3 (19.6–41.0)	27.1 (19.7–34.6)	28.3 (20.1–36.4)	33.8 (25.5–42.0)
Education level										
Below high school	35.6 (30.1–41.2)	32.5 (27.0–38.0)	29.2 (24.2–34.2)	29.2 (24.6–33.8)	27.6 (22.4–32.7)	27.5 (22.6–32.3)	24.1 (17.3–31.0)	18.9 (13.7–24.1)	18.5 (14.2–22.7)	16.6 (11.3–22.0)
High school graduate or GED	25.6 (18.7–32.4)	25.4 (18.7–32.1)	27.6 (22.5–32.7)	24.1 (19.6–28.6)	26.9 (22.5–31.4)	27.5 (24.2–30.8)	25.8 (17.1–34.5)	26.4 (22.2–30.6)	23.7 (19.1–28.4)	27.3 (21.0–33.5)
Some college or above	38.5 (33.9–43.1)	42.0 (35.7–48.4)	43.0 (37.7–48.2)	46.6 (39.4–53.8)	45.5 (38.1–52.8)	44.9 (39.1–50.8)	50.1 (44.2–56.0)	54.7 (48.7–60.7)	57.7 (51.6–63.8)	56.1 (47.7–64.6)
Marital status										
Married or living with partner	61.3 (55.0–67.7)	59.7 (54.9–64.4)	61.5 (55.8–67.2)	58.3 (53.3–63.2)	61.2 (56.4–66.0)	60.2 (54.0–66.4)	62.1 (54.8–69.5)	57.1 (51.3–62.9)	58.4 (50.4–66.4)	59.2 (52.7–65.7)
Never married	5.3 (2.9–7.6)	8.2 (4.5–11.8)	4.5 (2.5–6.5)	8.8 (5.9–11.8)	8.3 (5.2–11.4)	7.4 (5.1–9.7)	9.7 (3.9–15.6)	9.7 (7.3–12.2)	10.3 (7.2–13.5)	6.3 (4.0–8.5)
Widowed/divorced/separated	23.8 (17.6–30.0)	32.2 (26.5–37.8)	34.0 (29.3–38.8)	32.9 (27.6–38.1)	30.5 (26.1–34.8)	32.4 (27.2–37.7)	28.1 (22.7–33.6)	33.2 (27.9–38.5)	31.3 (24.1–38.4)	34.4 (28.8–39.9)
Employment status										
Unemployed	32.1 (27.7–36.5)	31.3 (25.9–36.6)	33.9 (29.2–38.7)	33.0 (28.1–37.9)	30.0 (23.9–36.0)	35.2 (30.3–40.2)	34.1 (27.2–41.0)	31.1 (26.5–35.6)	32.6 (26.5–38.6)	30.5 (24.1–36.9)
Employed	67.9 (63.5–72.3)	68.7 (63.4–74.1)	66.1 (61.3–70.8)	67.0 (62.1–71.9)	70.0 (64.0–76.1)	64.8 (59.8–69.7)	65.9 (59.0–72.8)	68.9 (64.2–73.5)	67.4 (61.4–73.5)	69.5 (63.1–75.9)

CI, confidence interval; GED, General Equivalent Diploma; NHANES, National Health and Nutrition Examination Survey; PIR, ratio of family income to poverty level.

^a^ Sample for each 2-year interval is unweighted, but all other numbers in the table are weighted percentages with 95% CI.

^b^ Includes Non-Hispanic Asian, other Hispanic, Multi-Racial, and other races.

### 3.2. Trends in unhealthy lifestyle factors in respondents with CVD

The proportion of respondents in each survey with unhealthy lifestyle factors is shown in Table 2 and the trends for change in Table 3.

**Table 2:** Trends in the age-adjusted^a^ prevalence of unhealthy lifestyle factors among adult NHANES respondents with cardiovascular disease.

Unhealthy lifestyle factors, % (95% CI)	NHANES survey										
	1999–2000	2001–2002	2003–2004	2005–2006	2007–2008	2009–2010	2011–2012	2013–2014	2015–2016	2017–2018	*p*
Current smoking status	22.7 (17.0–28.3)	23.7 (17.1–30.2)	24.2 (19.9–28.5)	23.0 (18.1–27.9)	19.2 (16.1–22.3)	21.0 (17.0–25.0)	25.4 (21.2–29.5)	21.1 (15.9–26.3)	23.2 (17.0–29.4)	21.8 (16.4–27.1)	0.833
Poor diet											
Primary AHA score <20	47.5 (37.9–57.0)	47.5 (37.9–57.0)	49.4 (42.5–56.3)	47.5 (41.5–53.4)	42.3 (37.5–47.2)	42.7 (35.6–49.9)	42.5 (36.9–48.0)	43.3 (36.6–50.0)	43.4 (38.4–48.4)	37.5 (25.7–49.3)	0.007
Secondary AHA score <32	34.0 (25.7–42.3)	35.6 (28.6–42.5)	36.8 (31.1–42.5)	32.8 (26.7–39.0)	34.7 (29.8–39.5)	30.4 (25.3–35.6)	27.4 (21.0–33.7)	30.9 (26.7–35.2)	33.2 (27.5–38.8)	31.8 (24.1–39.5)	0.060
Obesity	32.0 (27.2–36.8)	35.8 (30.7–40.8)	36.9 (32.9–40.8)	38.9 (34.2–43.5)	36.8 (30.9–42.6)	47.4 (44.3–50.5)	42.4 (36.0–48.8)	45.1 (40.3–49.8)	47.0 (41.3–52.7)	47.9 (39.9–55.8)	0.000
Physical inactivity^b^	NA	NA	NA	NA	50.1 (45.3–54.9)	52.3 (48.1–56.6)	50.0 (45.5–54.4)	58.0 (51.0–65.0)	46.8 (40.6–53.1)	43.0 (37.3–48.6)	0.377
Sedentary behavior^b^	NA	NA	NA	NA	38.3 (34.5–42.0)	39.0 (35.3–42.6)	44.4 (38.9–49.8)	62.1 (58.3–65.9)	47.3 (41.2–53.4)	38.1 (32.6–43.7)	0.719
Depression^c^	NA	NA	NA	7.0 (4.2–9.9)	10.1 (7.8–12.3)	11.6 (8.6–14.6)	9.9 (6.0–13.9)	15.3 (11.3–19.4)	10.4 (7.0–13.7)	13.9 (10.2–17.6)	0.056

AHA, American Heart Association; NHANES, National Health and Nutrition Examination Survey.

^a^ Direct age-standardization was achieved using three age groups (20–44, 45–64, and ≥65 years).

^b^ Data were available from 2007 through 2018.

^c^ Data were available from 2005 through 2018.

**Table 3:** Trends in unhealthy lifestyle factors in adult NHANES respondents with cardiovascular disease^a^.

Outcome	Inflection point	Annual percent change before inflection %/year (95% CI)	Annual percent change after inflection %/year (95% CI)
Physical inactivity	2013–2014	2.92 (−28.10–47.29)	−11.65 (−68.47–147.58)
Sedentary behavior	2013–2014	19.53 (−24.05–88.13)	−20.56 (−74.39–146.39)
		Average annual percentage change, %/year (95% CI)	
Current smoking status	—	−0.28 (−3.22 to 2.74)
Obesity	—	4.53 (2.15–5.97)^*^
Poor diet (primary AHA score <20)	—	−2.28 (−3.74 to 0.81)^*^
Poor diet (secondary AHA score <32)	—	−1.80 (−3.67 to 0.11)
Depression	—	8.48 (−0.35 to 18.08)

AHA, American Heart Association; CI, confidence interval; NHANES, National Health and Nutrition Examination Survey.

^a^ Direct age-standardization was achieved using three age groups (20–44, 45–64, and ≥65 years).

^*^ *p* < 0.05.

The current status of smoking showed no significant change over the 10 survey periods [1999–2000 (22.7%; 95% CI: 17.0%–28.3%) to 2017–2018 (21.8%; 16.4%–27.1%), AAPC: −0.28%, *p* = 0.833]. Poor diet showed a trend for decreasing prevalence. The proportion of respondents with a primary AHA score <20 reduced significantly from 47.5% (37.9–57.0) to 37.5% (25.7–49.3) between the 1999 and 2000 and the 2017–2018 surveys (AAPC: −2.28%, *p* = 0.007). The proportion of respondents with a secondary AHA score <32 also reduced, but not significantly, from 34% (25.7–42.3) to 31.8% (24.1–39.5; AAPC: −0.18%, *p* = 0.062). The mean secondary AHA score was 37.7 (35.9–39.5) in 1999–2000 and 39.1 (37.2–41.0) in 2017–2018 (an improvement of 3.7%; Table 4).

**Table 4:** Mean PHQ-9 and AHA secondary scores, BMI, physical activity, and sitting durations in adult NHANES respondents with cardiovascular disease for the period between 1999 and 2018.

Age-standardized mean (95% CI)	NHANES survey									
	1999–2000	2001–2002	2003–2004	2005–2006	2007–2008	2009–2010	2011–2012	2013–2014	2015–2016	2017–2018
PHQ-9 score	NA	NA	NA	2.9 (2.4–3.4)	3.8 (3.4–4.1)	3.6 (3.1–4.1)	3.7 (3.0–4.3)	4.3 (3.7–5.0)	4.1 (3.5–4.7)	4.4 (3.4–5.3)
Primary AHA score	20.4 (19.1–21.8)	20.0 (18.6–21.3)	20.7 (19.3–22.0)	21.2 (20.4–22.0)	21.7 (20.3–23.1)	21.4 (20.2–22.7)	21.4 (20.5–22.4)	21.7 (20.9–22.6)	21.1 (20.1–22.2)	23.1 (21.5–24.7)
Secondary AHA score	37.7 (35.9–39.5)	36.5 (34.5–38.5)	36.9 (35.3–38.6)	37.9 (36.6–39.2)	38.3 (36.5–40.0)	38.3 (36.5–40.1)	38.6 (36.8–40.3)	38.7 (36.9–40.4)	37.6 (35.9–39.3)	39.1 (37.2–41.0)
BMI (kg/m^2^)	28.4 (27.7–29.1)	29.4 (28.5–30.3)	29.1 (28.5–29.8)	29.5 (28.8–30.3)	29.6 (29.0–30.3)	30.5 (29.7–31.2)	29.7 (28.8–30.7)	30.4 (29.7–31.1)	30.7 (29.6–31.9)	30.8 (30.0–31.7)
Moderate physical activity duration (min/week)	NA	NA	NA	NA	388.6 (310.9–466.3)	348.9 (281.5–416.3)	303.3 (241.6–364.9)	256.6 (194.4–318.7)	363.2 (310.9–415.6)	396.9 (319.9–473.9)
Vigorous physical activity duration (min/weeks)	NA	NA	NA	NA	139.6 (97.4–181.9)	101.8 (71.3–132.4)	128.2 (68.2–188.3)	99.9 (51.6–148.1)	174.1 (124.0–224.3)	136.0 (85.3–186.7)
Sitting duration (h/day)	NA	NA	NA	NA	6.1 (5.8–6.4)	6.2 (5.9–6.4)	6.6 (6.2–6.9)	7.6 (7.4–7.9)	6.9 (6.6–7.3)	6.3 (5.9–6.7)

AHA, American Heart Association; BMI, body mass index; CI, confidence interval; NHANES, National Health and Nutrition Examination Survey; PHQ-9, Patient Health Questionnaire-9.

^a^ Direct age-standardization was achieved using three age groups (20–44, 45–64, and ≥65 years).

Despite showing trends for improvement in diet, the prevalence of obesity and depression increased over the course of the surveys. In line with mean BMI (Table 4), the obesity prevalence rate increased from 32.0% (27.2–36.8) in 1999–2000 to 47.9% (39.9–55.8) in 2017–2018 (AAPC: 4.53%, *p* = 0.002). The age-adjusted prevalence rate of depression increased non-significantly from 7% (4.2–9.9) in 2005–2006 to 13.9% (10.2–17.6) in 2017–2018 (AAPC: 8.48%, *p* = 0.056). The mean PHQ-9 score increased from 2.9 (2.4–3.4) in 2005–2006 to 4.4 (3.5–4.7) in 2017–2018, representing a 51.7% increase (Table 4).

The changes in physical inactivity and sedentary behavior prevalence were nonlinear, with an increasing trend between 2007 and 2013 (physical inactivity APC: 2.92%, *p* = 0.494; sedentary behavior APC: 19.53%, *p* = 0.126), an inflection point around 2013–2014, and a decreasing trend between 2015 and 2018 (physical inactivity APC: −11.65%, *p* = 0.369; sedentary behavior APC: −20.56%, *p* = 0.235). The age-adjusted prevalence rate of physical inactivity increased from 50.1% (45.3–54.9) in 2007–2008 to 58.0% (51.0–65.0) in 2013–2014 and then decreased to 43.0% (37.3–48.6) in the 2017–2018 survey. The respective prevalence rate of sedentary behavior was 38.3% (34.5%–42.0) in 2007–2008, 62.1% (58.3–65.9) in 2013–2014, and 38.1% (32.6–43.7) in the 2017–2018 survey. These findings were also reflected in the mean levels of moderate and vigorous physical activity and sitting duration (Table 4). The mean duration weekly of vigorous and moderate physical activity was the shortest, while the mean sedentary duration per day was the longest, in the 2013–2014 survey.

Trends in the prevalence of unhealthy lifestyle factors were similar after adjustment for age when 4-year survey cycles were used (Table S1 in the Supplementary material).

**Figure 1:**
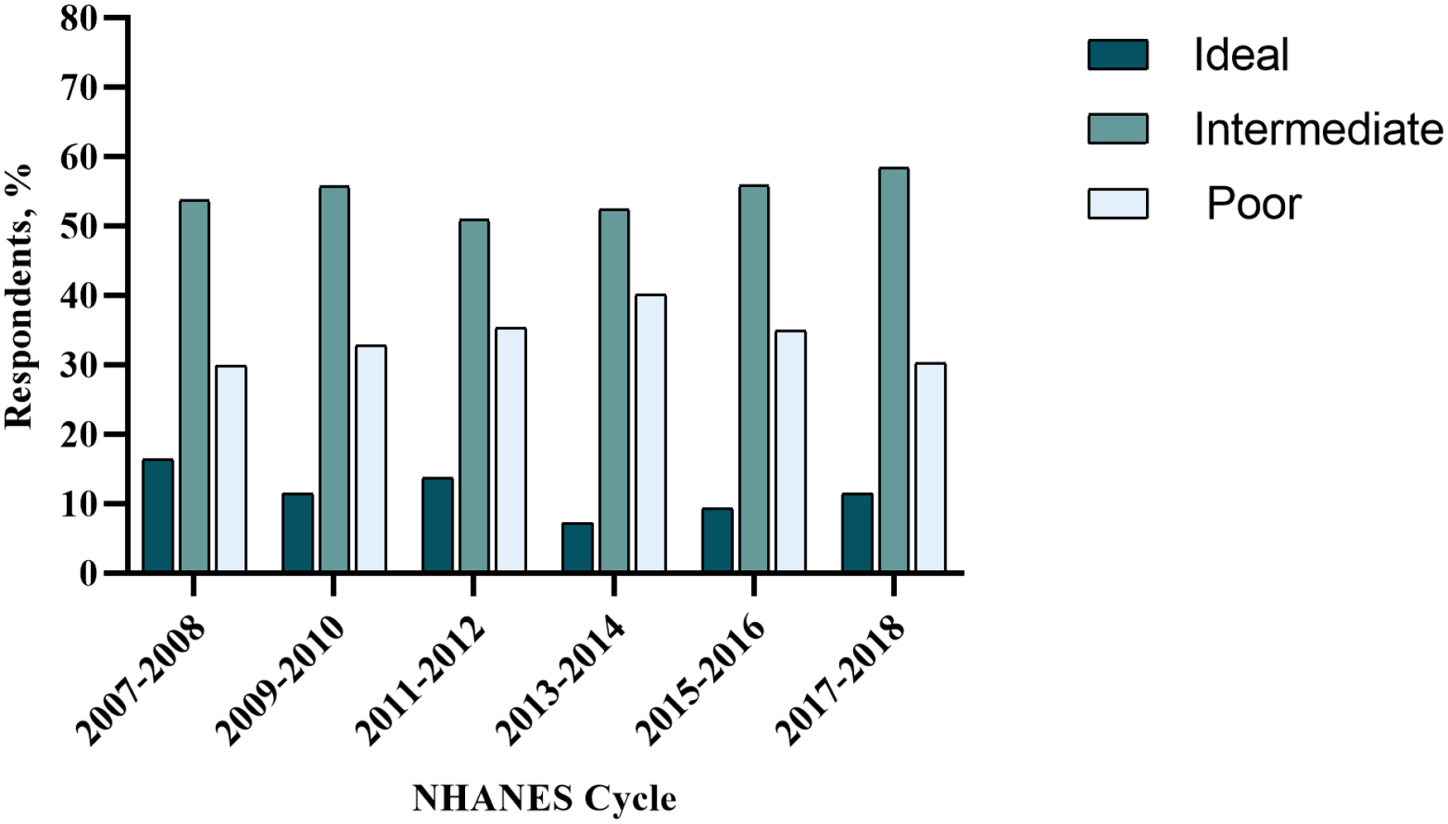
Unhealthy lifestyle score in adult NHANES respondents with cardiovascular disease for the period between 2007 and 2018. NHANES, National Health and Nutrition Examination Survey.

The prevalence score of unhealthy lifestyles during 2007–2018 is shown in Figure 1 and Supplementary Figure S1 and trends in the score of unhealthy lifestyles are shown in Table S2 in the Supplementary material. The prevalence rate of poor lifestyle increased significantly from 29.9% (26.4–33.4) in 2007–2008 to 40.1% (36.2–44.0) in 2013–2014 (APC: 10.22%, *p* = 0.037) and then decreased insignificantly to its original level in 2017–2018 [30.2% (26.4–34.0); APC: −12.75%, *p* = 0.053]. There was a modest but no significant decrease in the prevalence rate of ideal lifestyle (AAPC: −8.73%, *p* = 0.155). The prevalence rate of intermediate lifestyle remains stable over time (AAPC: 1.28%, *p* = 0.290).

### 3.3. Odds ratios between unhealthy lifestyles and sociodemographic characteristics

Odds ratios for each unhealthy lifestyle factor, adjusted for age, sex, and race/ethnicity, are presented in Supplementary Table S2. Full-adjusted ORs in Table 5 were adjusted by age, sex, race/ethnicity, family income, education level, marital status, and employment status. Compared with respondents aged ≧65 years, those who were younger age were more likely to have a poor diet but less likely to be physically inactive. Respondents aged 45–64 years were more likely to be obese than those aged 20–44 years. Female respondents were more likely to have depression and physical inactivity than male respondents but were less likely to be current smokers and have poor diet. Compared with non-Hispanic white respondents, non-Hispanic black respondents were more likely to be obese and physically inactive. We saw trends for increased likelihood of depression and smoking among respondents with a low income (PIR <1.30 vs. higher PIR). Respondents with lower educational attainment were more likely to be obese, physically inactive, and have poor diet. Compared with respondents who were married or living with their partner, those who were widowed, divorced, or separated were more likely to be current smokers, depressed, physically inactive, and sedentary. The risk of depression, obesity, and physical inactivity was higher among respondents who were unemployed compared with those who were employed.

**Table 5:** Full-adjusted^a^ odds ratio for unhealthy lifestyle factors in adult NHANES respondents with cardiovascular disease for the period between 1999 and 2018.

OR (95% CI)	Current smoking	Depression	Poor diet^b^	Obesity	Physical inactivity	Sedentary behavior
Age group						
20–44 years	1 (reference)	1 (reference)	1 (reference)	1 (reference)	1 (reference)	1 (reference)
45–64 years	0.88 (0.69–1.13)	1.42 (0.97–2.09)	0.64 (0.50–0.82)^*^	1.29 (1.00–1.67)^*^	1.61 (1.18–2.19)^*^	1.11 (0.84–1.48)
≥65 years	0.18 (0.13–0.24)^*^	0.46 (0.26–0.79)^*^	0.31 (0.24–0.40)^*^	0.76 (0.58–0.99)^*^	2.13 (1.56–2.89)^*^	1.23 (0.96–1.58)
Sex						
Female	1 (reference)	1 (reference)	1 (reference)	1 (reference)	1 (reference)	1 (reference)
Male	1.58 (1.30–1.92)^*^	0.57 (0.44–0.72)^*^	1.19 (1.01–1.39)^*^	0.91 (0.77–1.06)	0.72 (0.61–0.86)^*^	0.94 (0.79–1.12)
Race/ethnicity						
Non-Hispanic White	1 (reference)	1 (reference)	1 (reference)	1 (reference)	1 (reference)	1 (reference)
Non-Hispanic Black	0.85 (0.70–1.04)	0.92 (0.66–1.29)	0.96 (0.81–1.15)	1.53 (1.26–1.85)^*^	1.37 (1.09–1.73)^*^	0.96 (0.78–1.18)
Hispanic	0.43 (0.32–0.57)^*^	1.12 (0.80–1.57)	0.59 (0.47–0.75)^*^	1.04 (0.86–1.26)	1.02 (0.79–1.31)	0.51 (0.38–0.67)^*^
Other^c^	1.22 (0.77–1.95)	1.02 (0.63–1.65)	0.48 (0.35–0.65)^*^	0.57 (0.39–0.83)^*^	1.12 (0.82–1.52)	0.64 (0.46–0.89)^*^
Family income (PIR)						
<1.30	1 (reference)	1 (reference)	1 (reference)	1 (reference)	1 (reference)	1 (reference)
1.30–3.49	0.77 (0.60–0.97)^*^	0.67 (0.50–0.91)^*^	0.88 (0.74–1.05)	0.96 (0.81–1.13)	1.05 (0.88–1.25)	0.90 (0.71–1.15)
≥3.50	0.43 (0.33–0.56)^*^	0.35 (0.21–0.60)^*^	0.58 (0.46–0.72)^*^	0.78 (0.62–0.98)^*^	0.56 (0.43–0.72)^*^	1.20 (0.91–1.59)
Education level						
Below high school	1 (reference)	1 (reference)	1 (reference)	1 (reference)	1 (reference)	1 (reference)
High school graduate or GED	0.84 (0.69–1.03)	0.76 (0.55–1.05)	0.78 (0.63–0.96)^*^	1.35 (1.12–1.63)^*^	0.60 (0.48–0.75)^*^	0.99 (0.78–1.27)
Some college or above	0.58 (0.47–0.72)^*^	0.72 (0.52–1.00)	0.65 (0.53–0.78)^*^	1.22 (1.00–1.48)^*^	0.63 (0.50–0.79)^*^	1.35 (1.05–1.74)^*^
Marital status						
Married or living with partner	1 (reference)	1 (reference)	1 (reference)	1 (reference)	1 (reference)	1 (reference)
Never married	1.27 (0.97–1.67)	1.25 (0.72–2.17)	1.20 (0.91–1.58)	0.77 (0.56–1.06)	1.16 (0.83–1.62)	1.14 (0.81–1.60)
Widowed/divorced/separated	1.46 (1.21–1.77)^*^	1.49 (1.11–2.00)^*^	1.10 (0.92–1.32)	0.99 (0.84–1.17)	1.55 (1.25–1.92)^*^	1.49 (1.22–1.82)^*^
Employment status						
Employed	1 (reference)	1 (reference)	1 (reference)	1 (reference)	1 (reference)	1 (reference)
Unemployed	1.06 (0.83–1.37)	2.27 (1.46–3.52)^*^	0.98 (0.80–1.19)	1.30 (1.09–1.54)^*^	1.89 (1.47–2.45)^*^	0.99 (0.78–1.26)

GED, General Equivalent Diploma; NHANES, National Health and Nutrition Examination Survey; OR, odds ratio; PIR, ratio of family income to poverty level; CI, confidence interval.

^a^ Odds ratio with 95% confidence intervals were adjusted for age, sex, race/ethnicity, family income, education level, marital status, employment status group.

^b^ Poor diet was defined as achieving <32 for the secondary AHA score.

^c^ Includes Non-Hispanic Asian, other Hispanic, Multi-Racial, and other.

^*^ *p* < 0.05.

## 4. Discussion

In this analysis of serial cross-sectional NHANES surveys from 1999 through 2018, we found a reduction in the prevalence of poor diet (primary AHA score) among US adults with CVD, while the prevalence rates of current smoking, sedentary behavior, obesity, and depression either remained stable or increased. The prevalence rate of physical inactivity slightly reduced between 2007 and 2018, with no statistical significance. The proportion of poor lifestyle peaked in 2013–2014 and then decreased insignificantly between 2015 and 2018. These findings suggest that substantial clinical and policy measures targeting unhealthy lifestyles among adults with CVD are urgently required. Life's Essential 8 includes the eight components of cardiovascular health: healthy diet, participation in physical activity, avoidance of nicotine, healthy sleep, healthy weight, and healthy levels of blood lipids, blood glucose, and blood pressure (21). Previous studies (11–13, 21–23) have assessed the trends in CVD risk factors and metrics among the general NHANES respondents; however, few studies have examined these in patients with existing CVD. These patients may greatly benefit from lifestyle improvement to reduce morbidity and mortality.

Although tobacco control policies (including the US 2003 Framework Convention on Tobacco Control treaty and the “Tobacco 21” law) enforce bans on tobacco advertising, and increases in tobacco prices are ever more prohibitory for smoking, the percentage of current smokers shows no significant reduction between 1999 and 2018 in this study (21, 22), which is similar to a recent study in the US general population (13). Interventions are warranted to further disincentivize smoking, including paying special attention on populations who we found were more likely to smoke, including young males, those with low incomes, and those who were unemployed, widowed, divorced, or separated.

Paralleling a recent trend for improved diet in US adults (22), our research showed a small increase in the mean AHA score and a small decrease in the prevalence rate of poor diet from 1999 to 2018 in adults with CVD. These trends could be attributed to dietary guidelines published before the year 2000 that recommended a decreased intake of total fats and sugar (23). After 2000, progress in nutritional science, increased advocacy efforts, and updated dietary guidelines have consistently promoted the health benefits of fruits, vegetables, nuts/seeds, polyunsaturated fats, and the harm of sugar-sweetened beverages (SSBs) (24).

Despite the observed trends for improvements in diet, the age-adjusted prevalence of obesity and mean BMI increased in the last 2 decades in the US. Although the reasons for this are unclear, we found that non-Hispanic Black respondents, those aged 45–64 years, and those without employment were more likely to be obese, suggesting that health education and support should focus on helping these populations. There is an urgent need to identify the factors that drive the ever-increasing trend of obesity in the US, as well as to evaluate the effectiveness of current policies and programs designed to reduce obesity.

Consistent with earlier findings (25), we observed increasing prevalence rates of depression in US adults with CVD. Previous studies have reported a potential association between depression and CVD (10, 26). This highlights the need for specific interventions for depression in patients with CVD. We found that respondents with low education and who were female, widowed/divorced/separated, or unemployed were more likely than comparator groups to have depression, which should be paid more attention to.

The age-adjusted prevalence rates of physical inactivity and sedentary behavior increased during the 2007 to 2014 period and then decreased over time. Overall, a slight but not significant reduction in the prevalence rate of physical inactivity was found during 2017–2018 compared with 2007–2008. The prevalence rate of sedentary behavior increased insignificantly from 2007 to 2014 and then remained stable. Mean sitting duration was the longest, and physical activity duration was the shortest, in the 2013–2014 survey. These findings were broadly consistent with a recent analysis showing that the proportion of adherence to the PAG for aerobic activity among US adults with CVD from 2007 to 08 to 2017–18 had increased (15). Findings to date suggest that physical inactivity in US adults with CVD has improved since the release of the PAG for Americans in 2008. A declining prevalence of sedentary behavior was not observed over time. We found that respondents with a college degree or more education and who are widowed/divorced/separated were more likely than comparator groups to be sedentary. Further nationwide efforts to encourage physical activity and reduce sedentary time would help improve health in people with CVD in the US.

### 4.1. Strengths and limitations

The strengths of our study are the large sample size, nationally representative data, rigorous sampling design, and the reliability of study measurements. Several limitations also apply. First, NHANES is a cross-sectional study, with different respondents in each survey. This limits our ability to look at longitudinal change at the individual level. Second, NHANES response rates declined over time; however, the data had weighting adjustment to non-response bias. The sampling weights were used in our analysis according to NHANES analytic guidelines. Third, because our definition of CVD relied on self-reported data, there was a possibility of misdiagnosis. Further, self-reported data on diet and physical activity could have recall bias. Fourth, questionnaires on physical activity, sedentary time, and depression assessment changed between 1999 and 2018. Therefore, we examined the change in trends over the past 10 years. Finally, biomarker levels for blood lipids, fasting plasma glucose, and blood pressure were not included in this analysis; these would have proved informative to indicate CVD risk.

## 5. Conclusions

There is a significant reduction in the prevalence rate of poor diet among US adults with CVD between 1999 and 2018, while the prevalence rate of obesity showed increasing trends over this period. A non-statistically significant decrease in the prevalence of physical inactivity was observed. The prevalence of current smoking status, sedentary behavior, and depression was either stable or showed an insignificant increase.

## Data Availability

The original contributions presented in the study are included in the article/Supplementary Material, further inquiries can be directed to the corresponding authors.
