# Supplementary material for: Trends in unhealthy lifestyle factors in US NHANES respondents with cardiovascular disease for the period between 1999 and 2018
Source: Front Cardiovasc Med. 2023 May 19;10:1169036. doi: 10.3389/fcvm.2023.1169036 (PMC10235541; doi:10.3389/fcvm.2023.1169036)

Supplemental Online Content

eTable 1: Prevalence of Unhealthy Lifestyle Factors in Adult NHANES Respondents with Cardiovascular Disease from 1999 to 2018 (4-year cycles)

eTable 2: Trends in Unhealthy Lifestyles Score Unhealthy Lifestyle Factors in Adult NHANES Respondents with Cardiovascular Disease from 2007 to 2018.

eTable 3: Adjusted Odds Ratio for Unhealthy lifestyle factors in Adult NHANES Respondents with Cardiovascular Disease from 1999 to 2018.

eFigure 1: Prevalence of Unhealthy Lifestyles Score in Adult NHANES Respondents with Cardiovascular Disease from 2007 to 2018.

eTable 1: Prevalence of Unhealthy Lifestyle Factors in Adult NHANES Respondents with Cardiovascular Disease from 1999 to 2018 (4-year cycles)[a].

Unhealthy lifestyle factors, % (95%CI)	NHANES survey (4-year cycles)				
	1999-2002	2003-2006	2007-2010	2011-2014	2015-2018
Current Smoking	23.2 (18.9-27.4)	23.6 (20.4-26.9)	20.1 (17.5-22.6)	23.1 (19.7-26.5)	22.5 (18.4-26.6)
Depression	NA	NA	10.0 (8.3-11.7)	12.7 (9.9-15.6)	12.1 (9.6-14.6)
Poor diet (secondary AHA score <32)	34.8 (29.4-40.2)	35.0 (30.7-39.3)	32.7 (29.1-36.3)	29.3 (25.4-33.1)	32.5 (27.9-37.2)
Obesity	33.9 (30.4-37.4)	37.8 (34.8-40.9)	41.9 (38.5-45.2)	43.8 (39.8-47.8)	47.4 (42.6-52.3)
Physical inactivity	NA	NA	51.2 (48.0-54.3)	54.2 (49.8-58.5)	44.9 (40.7-49.2)
Sedentary behavior	NA	NA	38.6 (36.0-41.2)	53.6 (50.2-57.0)	42.8 (38.4-47.2)

[a] Direct age-standardization was achieved using three age groups (20-44, 45-64, and ≥65 years). CI, confidence interval; NHANES, National Health and Nutrition Examination Survey.

eTable 2: Trends in Unhealthy Lifestyles Score in Adult NHANES Respondents with Cardiovascular Disease from 2007 to 2018. [a]

Outcome	Inflection point	Annual percent change before inflection<br><br>%/year (95% CI)	Annual percent change after inflection<br><br>%/year (95% CI)
Poor Lifestyle	2013-2014	10.22 (2.54-18.48)*	-12.73 (-24.47-0.82)
		**Average annual percentage change, %/year (95% CI)**	
Intermediate Lifestyle	-	1.28 (-1.62-4.27)	
Ideal Lifestyle	-	-8.73 (-21.02-5.48)	

[a] Direct age-standardization was achieved using three age groups (20-44, 45-64, and ≥65 years). CI, confidence interval; NHANES, National Health and Nutrition Examination Survey,

* $p < 0.05$.

eTable3: Adjusted[a] Odds Ratio for Unhealthy lifestyle factors in Adult NHANES Respondents with Cardiovascular Disease from 1999 to 2018.

OR(95% CI)	Current smoking	Depression	Poor diet[c]	Obesity	Physical inactivity	Sedentary behavior
**Age group**						
20-44 years	1(reference)	1(reference)	1(reference)	1(reference)	1(reference)	1(reference)
45-64 years	0.89(0.72-1.10)	1.65(1.20-2.28)*	0.63(0.50-0.79)*	1.38(1.08-1.76)*	1.87(1.39-2.52)*	1.14(0.85-1.54)
≥65 years	0.23(0.18-0.29)*	0.79(0.52-1.19)	0.33(0.27-0.40)*	0.91(0.74-1.13)	3.41(2.55-4.56)*	1.24(0.99-1.55)
**Sex**						
Female	1(reference)	1(reference)	1(reference)	1(reference)	1(reference)	1(reference)
male	1.32(1.09-1.60)*	0.47(0.37-0.59)*	1.10(0.94-1.28)	0.87(0.75-1.01)	0.60(0.51-0.71)*	0.89(0.76-1.06)
**Race/ethnicity**						
Non-Hispanic White	1(reference)	1(reference)	1(reference)	1(reference)	1(reference)	1(reference)
Non-Hispanic Black	1.16(0.95-1.41)	1.38(1.02-1.87)*	1.17(0.99-1.39)	1.52(1.26-1.83)*	1.84(1.48-2.30)*	0.91(0.75-1.11)
Hispanic	0.61(0.46-0.81)*	1.58(1.16-2.14)*	0.76(0.62-0.94)*	1.00(0.83-1.22)	1.39(1.11-1.73)*	0.44(0.34-0.57)*
Other [b]	1.25(0.80-1.94)	1.13(0.70-1.82)	0.50(0.37-0.68)*	0.57(0.39-0.83)	1.19(0.85-1.66)	0.59(0.42-0.82)*
**Family income(PIR)**						
<1.30	1(reference)	1(reference)	1(reference)	1(reference)	1(reference)	1(reference)
1.30-3.49	0.65(0.51-0.83)*	0.52(0.39-0.68)*	0.77(0.64-0.92)*	0.96(0.81-1.14)	0.80(0.66-0.96)*	0.89(0.70-1.13)
≥3.50	0.32(0.25-0.40)*	0.21(0.13-0.35)*	0.48(0.39-0.59)*	0.77(0.62-0.95)*	0.35(0.28-0.44)*	1.22(0.94-1.58)
**Education level**						
Below high school	1(reference)	1(reference)	1(reference)	1(reference)	1(reference)	1(reference)
High school graduate or GED	0.74(0.61-0.90)*	0.62(0.45-0.85)*	0.73(0.59-0.90)*	1.29(1.07-1.55)*	0.52(0.41-0.66)*	0.99(0.78-1.25)
Some college or above	0.45(0.36-0.55)*	0.46(0.33-0.63)*	0.55(0.45-0.66)*	1.08(0.90-1.28)	0.45(0.36-0.56)*	1.39(1.10-1.76)*
**Marital status**						
Married or living with partner	1(reference)	1(reference)	1(reference)	1(reference)	1(reference)	1(reference)
Never married	1.34(1.03-1.74)*	1.36(0.80-2.31)	1.24(0.95-1.62)	0.80(0.59-1.09)	1.19(0.87-1.63)	1.13(0.81-1.59)
Widowed/divorced/separated	1.75(1.45-2.12)*	1.89(1.41-2.53)*	1.25(1.04-1.50)*	1.03(0.87-1.22)	1.85(1.50-2.28)*	1.38(1.12-1.68)*

continued

OR(95% CI)	Current smoking	Depression	Poor diet	Obesity	Physical inactivity	Sedentary behavior
**Employment status**						
Employed	1(reference)	1(reference)	1(reference)	1(reference)	1(reference)	1(reference)
Unemployed	1.53(1.21-1.94)*	3.21(2.11-4.90)*	1.26(1.03-1.55)*	1.33(1.12-1.59)*	2.34(1.83-3.00)*	0.94(0.74-1.19)

[a] Odds ratio with 95% confidence intervals was adjusted for age, sex, and race/ethnicity group. [b] Includes Non-Hispanic Asian, other Hispanic, Multi-Racial, and other races. [c] Poor diet was defined as achieving <32 for the secondary AHA score; CI, confidence interval; GED, General Equivalent Diploma; NHANES, National Health and Nutrition Examination Survey; OR, odds ratio; PIR, ratio of family income to poverty level. * $p < 0.05$.

Figure 1: Prevalence of Unhealthy Lifestyles Score in Adult NHANES Respondents with Cardiovascular Disease from 2007 to 2018.

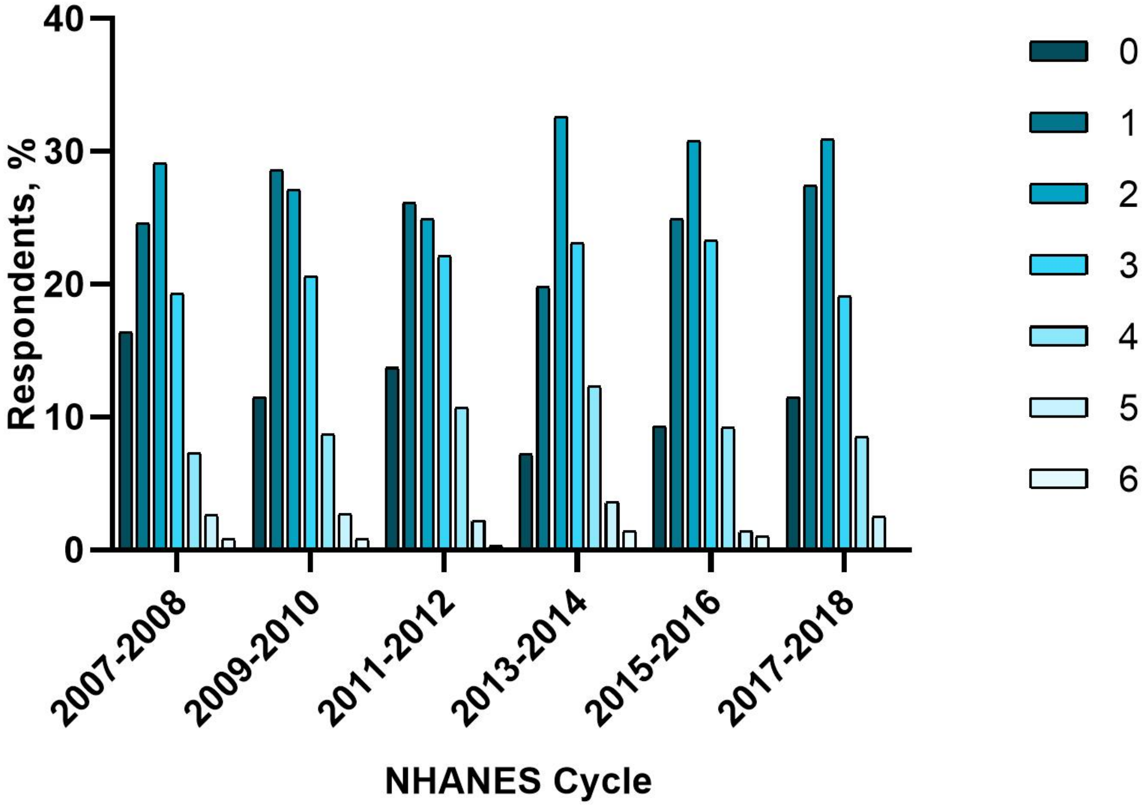

Supplement: Supplementary file 1 [file Datasheet1.pdf]
